# Defense and subversion: multilevel mitochondrial quality control and pathway‑specific mitophagy outcomes in *Mycobacterium tuberculosis* infection

**DOI:** 10.3389/fimmu.2026.2003142

**Published:** 2026-10-15

**Authors:** Xing Lv, Ping Xu

**Affiliations:** 1 Suzhou Medical College of Soochow University, Suzhou, Jiangsu, China; 2 Clinical Laboratory Center, Affiliated Infectious Diseases Hospital of Soochow University (Suzhou Fifth People’s Hospital), Suzhou, Jiangsu, China

**Keywords:** mitochondria, mitochondrial damage, mitochondrial quality control, mitophagy, *Mycobacterium tuberculosis*

## Abstract

Tuberculosis, caused by *Mycobacterium tuberculosis*, remains a leading cause of infectious mortality worldwide, a burden compounded by rising drug resistance. While mitochondria were long dismissed as mere powerhouses, they now function as hubs that integrate immune metabolism, innate immune signaling, and cell-death decisions. During infection, *M.tb* severely disrupts these hubs through organelle swelling, cristae remodeling, mitochondrial DNA release, and rewired oxidative phosphorylation. Despite this disruption, the field remains divided over whether mitophagy, the terminal tier of quality control, protects the host or serves the pathogen. The answer depends on the pathway engaged. Here, we synthesize evidence that *M.tb* engages a multilevel mitochondrial quality control network spanning chaperones and proteases, the integrated stress response, fusion and fission dynamics, and mitophagy. We propose that distinct mitophagy pathways yield mechanistically distinct, context-dependent outcomes during mycobacterial infection. Notably, the PINK1 and Parkin axis mediates both protective antibacterial xenophagy and pathogen-exploited mitophagy. Beyond this axis, BNIP3, NIX, and FUNDC1 can limit or promote bacterial survival, depending on pathway identity, infection stage, and host-cell metabolic state. This framework offers a way to interpret effects that appear to conflict, whether they protect the host or favor the pathogen, traces the outcome to which pathway is engaged, the context of infection, and the state of the host cell, and identifies specific MQC nodes, including mitochondrial reactive oxygen species thresholds and the PINK1-Parkin axis, as candidate targets for host-directed therapy.

## 1 Introduction

Tuberculosis (TB), caused by *Mycobacterium tuberculosis* (*M.tb*), remains one of the world’s deadliest infectious diseases and the leading cause of death attributable to a single infectious pathogen. *M.tb* is the classic example of an intracellular pathogen. Its success depends on its ability to survive and proliferate within host macrophages, the cells normally responsible for eliminating phagocytosed microorganisms. Understanding the host pathways that *M.tb* manipulates to establish its intracellular niche is essential for developing new therapeutic strategies, particularly amid the ongoing spread of drug-resistant TB.

Mitochondria have traditionally been regarded as the cell’s powerhouses, with ATP production through oxidative phosphorylation (OXPHOS) as their primary function. They are now well established, however, as central signaling hubs that coordinate immunometabolism, innate immune signaling, and regulated cell death (1–3). In addition, mitochondria generate reactive oxygen species (mtROS), which act as both bactericidal effectors and signaling molecules (4); and they release damage-associated molecular patterns (DAMPs), such as mitochondrial DNA (mtDNA), that are recognized by innate immune sensors (5); These organelles also provide the metabolic plasticity required for macrophages to polarize between pro-inflammatory (M1) and anti-inflammatory (M2) states (6). This expanded view of mitochondrial function is directly relevant to the understanding of intracellular infections. If mitochondria are central to metabolism, immunity, and cell death, pathogen-induced mitochondrial dysfunction is likely to have wide-ranging consequences for host defense.

To counteract the adverse effects on mitochondria caused by events such as intracellular infections, cells deploy a multilevel mitochondrial quality control (MQC) network that operates synergistically at the molecular, organellar, and cellular scales (7–9). At the molecular level, chaperones and proteases maintain protein homeostasis. The integrated stress response (ISR) provides early metabolic and translational buffering (10); mitochondrial fusion, fission, and biogenesis dynamically reshape the mitochondrial network (11); When damage exceeds repair capacity, mitophagy, the selective autophagy of mitochondria, irreversibly clears damaged organelles (12). Each of these levels is a potential site for host-pathogen interactions, yet no systematic review has explored how *M.tb* exploits the MQC levels as an integrated defense network.

Notably, mitophagy has emerged as a paradoxical player in TB. Activated PINK1-Parkin-mediated mitophagy to compete with xenophagy for shared autophagic machinery (13); whereas BNIP3-mediated mitophagy paradoxically benefits *M.tb* by suppressing cytokine production (14). Whether mitophagy is host-protective or host-damaging in TB therefore remains unresolved.

Several recent reviews have covered parts of the interaction between mitochondria and mycobacterial infection. Patrick and Watson (15) summarized mitochondria-dependent innate immune responses during *M.tb* infection, including mitochondrial damage, cytosolic DNA sensing, inflammasome activation, and metabolic remodeling. A broader review of bacterial infection examined the dual role of mitophagy, as a host-protective response and as a process exploited by intracellular pathogens (16). TB-focused reviews have since taken up metabolic and cell-death reprogramming during infection (17), mitophagy-associated signaling and host-directed therapeutic opportunities (18), and macrophage mitochondrial metabolism within a biochemical threshold model involving effectors such as ESAT-6, PtpA, and TNT (19). MQC across proteostasis, dynamics, biogenesis, and mitophagy has likewise been mapped in health and disease (20).

In this review, we aim to organize TB-associated mitochondrial perturbations within a multilevel MQC framework and to compare how distinct mitophagy pathways, including PINK1-Parkin, BNIP3/NIX, and FUNDC1, relate to xenophagy, mitochondrial ROS signaling, inflammatory output, and mycobacterial survival. This pathway-centered organization is intended as a conceptual framework for reconciling apparently divergent observations.

## 2 Mitochondrial quality control

### 2.1 Concept and components of mitochondrial quality control

Mitochondrial functional integrity depends on a multilevel, finely regulated MQC network. This network operates synergistically at the molecular, organellar, cellular, and even tissue levels to continuously monitor and repair mitochondrial damage or, when damage is irreversible, to selectively clear damaged mitochondria and thereby maintain the functional homeostasis of the mitochondrial population (1, 7). For this review, the MQC network is organized into four tiers (**Figure 1**). I. Molecular-level quality control relies on mitochondrial chaperones, endogenous proteases, and the ubiquitin-proteasome system to clear misfolded or damaged proteins (21, 22); II. The ISR and metabolic buffering operate via the OMA1-DELE1-HRI-eIF2α-ATF4 signaling axis, through which cells execute rapid translation attenuation and metabolic reprogramming to reduce the protein-folding burden on stressed mitochondria and reallocate metabolic resources, thereby buying time for repair (10, 23); III. Network dynamics remodeling and biogenesis: through continuous mitochondrial fusion and fission, damage is diluted, contents are mixed, and functional complementarity is achieved. When network function declines, PGC-1α drives the synthesis of new mitochondria to replenish the functional pool (24, 25); IV. Organelle-level selective clearance: when localized damage exceeds the repair capacity of tiers I–III, the damaged segments isolated by mitochondrial fission are delivered to lysosomes for degradation via the mitophagy pathway (26). Classic pathways include the PTEN induced putative kinase 1 (PINK1)-Parkin-mediated ubiquitin-dependent mechanism and the non-ubiquitin-dependent mechanisms mediated by receptors such as BCL2 Interacting Protein 3 (BNIP3)/NIP3-like protein X (NIX) and FUN14 domain-containing protein 1 (FUNDC1). These tiers are coupled through metabolic sensors and stress signaling networks, so that the response stays proportional. Mild stress triggers reversible buffering (tiers I–III), whereas severe or persistent stress leads to the irreversible degradation of mitochondria (tier IV).

**Figure 1:**
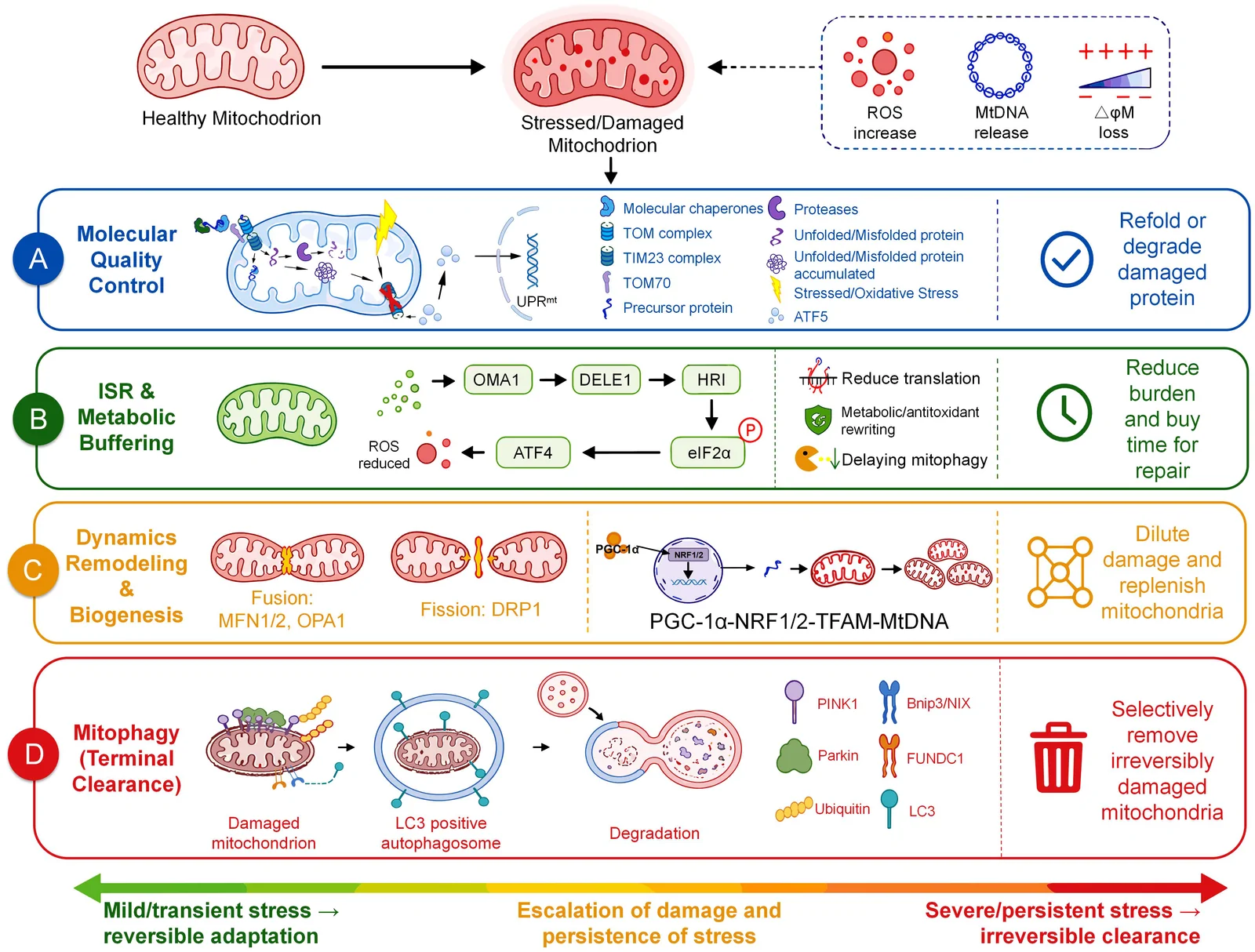
Multilevel mitochondrial quality control across escalating mitochondrial stress. MQC coordinates repair and clearance according to stress severity. **(A)** Molecular quality control maintains proteostasis through chaperones, protein import machinery, proteases, and the UPR^mt^. **(B)** The OMA1–DELE1–HRI–eIF2α–ATF4 axis reduces translational and metabolic burden, providing time for repair. **(C)** Mitochondrial fusion, fission, and PGC-1α–NRF1/2–TFAM-mediated biogenesis remodel the mitochondrial network and replenish functional mitochondria. **(D)** When damage becomes irreversible, PINK1–Parkin-, BNIP3/NIX-, and FUNDC1-mediated mitophagy selectively removes dysfunctional mitochondria. Thus, mild stress favors reversible adaptation, whereas severe or persistent stress promotes terminal mitochondrial clearance. mtROS, mitochondrial reactive oxygen species; mtDNA, mitochondrial DNA; ΔΨm, mitochondrial membrane potential; UPR^mt^, mitochondrial unfolded protein response; TOM70, translocase of the outer mitochondrial membrane 70; TIM23, translocase of the inner mitochondrial membrane 23; ATF5, activating transcription factor 5; ISR, integrated stress response; OMA1, OMA1 zinc metallopeptidase; DELE1, DAP3-binding cell death enhancer 1; HRI, heme-regulated inhibitor kinase; eIF2α, eukaryotic translation initiation factor 2α; ATF4, activating transcription factor 4; MFN1/2, mitofusin 1/2; OPA1, optic atrophy 1; DRP1, dynamin-related protein 1; PGC-1α, peroxisome proliferator-activated receptor gamma coactivator 1α; NRF1/2, nuclear respiratory factor 1/2; TFAM, mitochondrial transcription factor A; OXPHOS, oxidative phosphorylation; ATP, adenosine triphosphate; PINK1, PTEN-induced kinase 1; BNIP3, BCL2-interacting protein 3; NIX (BNIP3L), BCL2-interacting protein 3-like; FUNDC1, FUN14 domain-containing protein 1; LC3, microtubule-associated protein 1 light chain 3; Ub, ubiquitin.

### 2.2 Molecular-level quality control: chaperones, proteases, and the UPR^mt^

The maintenance of mitochondrial protein homeostasis, the first quality control checkpoint, begins before precursor proteins even enter the mitochondria. The cytosolic chaperones Heat shock protein (Hsp) 70/Hsp90 deliver nuclear-encoded mitochondrial precursor proteins to the outer-membrane receptor Tom70, holding them in a transport-competent state (27). Subsequently, the import motor, composed of mt-Hsp70 on the matrix side and translocase of inner mitochondrial membrane (TIM) 44/membrane import machinery protein (MIM)44, drives the transmembrane transport of TIM23 pathway substrates, pulling peptides unidirectionally into the mitochondrial matrix (28). Protein import therefore doubles as the first checkpoint of quality control. Under cellular stress, stress-induced degradation of TIM17A actively reduces the import capacity of the TIM23 complex, minimizing the burden that newly imported proteins would impose on damaged mitochondria (29). In budding yeast, impaired mitochondrial protein import activates the mitoCPR surveillance pathway. MitoCPR clears precursor proteins stalled at mitochondrial translocases and targets them for proteasomal degradation, thereby maintaining import and proteostasis (30).

Once inside the mitochondria, proteins must adopt their native conformation to function. The matrix chaperone Hsp60 and its co-chaperone Hsp10 are essential for the folding and oligomeric assembly of newly imported proteins (31, 32). The chaperone system does more than respond passively to stress. It constitutes a fundamental quality control mechanism for the maturation of newly imported proteins, determining whether they can be effectively integrated into the mitochondrial functional network. Using cryo-electron microscopy, Ehses et al. (33) showed that stress triggers matrix protein aggregation, cristae remodeling, and a reduction in ribosome number, and drives Hsp60 conformational rearrangement and Hsp60:Hsp10 complex assembly, revealing the structural basis of stress-induced remodeling of the mitochondrial protein biosynthetic machinery.

Imported mitochondrial proteins can fail to adopt the correct conformation or become damaged. When this happens, protease-mediated degradation serves as a critical line of defense for protein homeostasis. In the mitochondrial matrix, Lon peptidase 1(LONP1) is regarded as the central protease that suppresses the aggregation of misfolded proteins (34). It degrades misfolded substrates directly and also acts as a “gatekeeper” for newly imported matrix proteins (35). Meanwhile, the inner-membrane proteases YME1 Like 1 ATPase (YME1L) and OMA1 zinc metallopeptidase (OMA1) regulate the homeostasis and adaptability of mitochondrial inner-membrane proteins through stress-dependent proteolytic remodeling (36, 37). The mitochondrial protease network carries out terminal degradation, but through compartmentalized functions and reprogramming, it also determines whether damaged proteins are repaired and reused or cleared and eliminated.

When the capacity of local chaperones and proteases is exceeded, the mitochondrial unfolded protein response (UPR^mt^) transmits retrograde signals to the nucleus, orchestrating broader protective transcriptional programs. The UPR^mt^ was initially recognized through the observation that loss of mitochondrial genome function selectively induces the expression of mitochondrial chaperones (38). The canonical mechanistic framework was defined largely in *Caenorhabditis elegans* (39). Subsequent mechanistic studies identified the protease Caseinolytic peptidase P (ClpP) as a mediator of signal transduction from mitochondrial protein toxicity to the nuclear response (40), and showed that the activating transcription factor associated with stress-1 (ATFS-1) senses reduced mitochondrial import efficiency. Under basal conditions, ATFS-1 is continuously imported into mitochondria and degraded, whereas during stress, import is reduced and ATFS-1 redistributes to the nucleus to activate protective gene expression (39, 41). Mammalian and human cells retain part of this regulatory logic but use a more complex transcriptional network. Fiorese et al. (42) identified ATF5 as a functional mammalian analogue of ATFS-1. In HEK293T cells, ATF5 was required for induction of mitochondrial proteostasis genes and promoted recovery of mitochondrial function after stress. Recent clinical evidence from human tissue supports a role for UPR^mt^-mediated proteostasis in mitochondrial stress responses. In patients with heart failure, myocardial expression of several UPR^mt^-associated chaperones and proteases is reduced, and lower expression is associated with earlier disease progression (43). Zhang et al. (44) demonstrated that extracellular matrix remodeling triggers TGF-β signaling, inducing mitochondrial fission and UPR^mt^ activation, revealing that the UPR^mt^ can also be activated by extracellular signals. Thus, the UPR^mt^ is a transcriptional reprogramming checkpoint (**Figure 1A**). It links molecular QC to the broader stress responses discussed in the next section.

### 2.3 Integrated stress responses and metabolic buffering

However, mammalian mitochondrial stress does not invariably activate an ATF5-dominated program. Multi-omics analyses showed that several forms of mitochondrial dysfunction instead preferentially activate an ATF4-dependent ISR, with metabolic remodeling and translational adaptation occurring without robust induction of canonical ATF5-associated UPR^mt^ genes (45). Before committing damaged mitochondria to irreversible degradation, cells deploy reversible buffering strategies that reduce the burden on stressed organelles and create a window for repair. The ISR is the central coordinator of this early adaptation. Under mitochondrial stress, the inner-membrane protease OMA1 cleaves DAP3-binding cell death enhancer 1 (DELE1), and the resulting C-terminal fragment translocates to the cytoplasm, where it activates the heme-regulated inhibitor (HRI) kinase. HRI phosphorylates eIF2α, leading to global translation attenuation and selective translation of ATF4, which drives a broad range of cell-protective transcriptional programs (10, 23). This pathway suppresses overall translation, reducing the influx of newly synthesized proteins and the protein-folding burden, while simultaneously inducing ATF4-dependent expression of cell-protective genes to promote the remodeling of metabolic and antioxidant programs (45).

Following impairment of the mitochondrial respiratory chain, cells also undergo ATF4-dependent remodeling of one-carbon metabolism, serine biosynthesis, and transsulfuration pathways. These changes redistribute carbon flux and sulfur metabolism resources when OXPHOS efficiency declines, thereby maintaining nucleotide synthesis and the supply of reducing power (46). eIF2α signaling also promotes cysteine uptake and glutathione synthesis, reduces ROS levels, and improves cellular tolerance under hypoxia and stress (47). The ISR is more than a simple “shutdown”. It buys time for mitochondrial repair through temporary downregulation and resource reallocation (**Figure 1B**).

A recent discovery directly links the ISR to the regulation of mitophagy. By reducing overall protein synthesis, the ISR maintains the efficiency of mitochondrial precursor protein import and thereby inhibits PINK1-mediated mitophagy in an ATF4-independent manner, a regulation that operates only under mild mitochondrial stress and is lost under severe stress (48). Mitochondrial import motors can themselves sense protein misfolding and respond to disequilibrium by reducing import capacity. mitophagy is engaged only when this buffering is insufficient to maintain homeostasis (49). Stress-induced mitochondrial hyperfusion (SIMH) increases ATP production and pro-survival signaling (50), whereas starvation-induced mitochondrial elongation allows mitochondria to temporarily evade autophagic degradation and maintain cell viability (51). These mechanisms together reduce the load, preserve function, and delay clearance, maximizing opportunities for repair before degradation ensues.

### 2.4 Mitochondrial dynamics: fusion, fission, and biogenesis

The mitochondrial network is not composed of isolated organelles. Continuous fusion events enable the exchange of proteins, lipids, metabolic intermediates, and mtDNA-related components between mitochondria, thereby facilitating content mixing and functional complementarity (52, 53). At the molecular level, outer-membrane fusion is mediated by Mitofusin 1/2 (Mfn1/2), whereas inner-membrane fusion is driven by Optic Atrophy 1 (OPA1). The two processes act synergistically to maintain network connectivity. Studies of Mfn1/Mfn2 deficiency indicate that fusion is essential for maintaining mtDNA and respiratory chain activity (54). The fusion process is subject to precise, multi-level regulation. Even in the absence of exogenous stress, Parkin can inhibit outer-membrane fusion by ubiquitinating and degrading Mfn1/Mfn2, while OMA1 controls inner-membrane fusion through stress-dependent cleavage of OPA1 (55), These elements constitute a synergistic “dual-gate” mechanism spanning the inner and outer membranes (**Figure 1C**).

Mitochondrial fission mirrors the functional complementarity mediated by fusion and is also an essential step in quality control that dilutes damage and determines fate, rather than a mere sign of network disintegration (24). The classic study by Twig et al. (25) demonstrated that mitochondria can generate functionally asymmetric progeny during a continuous fusion/fission cycle. Progeny with higher membrane potential (ΔΨm) tend to reintegrate into the network, whereas those with decreased ΔΨm are more likely to be isolated and enter subsequent clearance processes. Network dynamics thus switch between “sharing” and “sorting” to maximize the retention of still-functional mitochondrial units, rather than simply maintaining morphological equilibrium. Furthermore, AMPK-mediated energy-stress-induced fission shows that under metabolic stress, fission can be actively mobilized as a structural remodeling strategy that rapidly adjusts network organization and limits the spread of localized damage (56). The inner mitochondrial membrane protein mitochondrial fission process 1 (MTFP1) can isolate damaged inner-membrane subregions by inhibiting inner-membrane fusion, and these subregions are subsequently sorted into small mitochondria via peripheral fission and degraded through mitophagy, thereby maintaining homeostasis at the mtDNA level (57). Fusion and fission are thus complementary. Working together in the service of mitochondrial quality control, fusion contributes to homeostasis through content sharing, functional complementarity, and damage buffering, beyond its role in maintaining morphology (**Figure 1C**).

When the overall functional capacity of the mitochondrial network declines beyond what fusion and fission can compensate for, cells deploy mitochondrial biogenesis to replenish the functional pool. Peroxisome proliferator-activated receptor gamma coactivator -1α (PGC-1α), a central co-activator that responds to energy demands and environmental changes, upregulates respiratory chain-related genes and promotes mitochondrial proliferation (58). By synergizing with nuclear transcription factors such as the nuclear respiratory factors Nuclear Respiratory Factor (NRF) -1 and NRF-2, PGC-1α drives the expression of main effectors such as mitochondrial transcription factor A (TFAM), thereby coupling the nuclear gene transcription program with mtDNA replication and transcription (59–61). Mitochondrial biogenesis is therefore a core compensatory mechanism. It restores oxidative metabolic capacity through the PGC-1α–NRF–TFAM axis once the mitochondrial network has functionally declined, rather than a simple increase in mitochondrial number (**Figure 1C**).

Recently, the research paradigm has shifted from “PGC-1α-driven mitochondrial proliferation” to an integrated network that links “biogenesis, transport, and quality control.” Mitochondrial biogenesis depends on more than local, passive initiation. Retrograde transport of mitochondria from the distal ends of axons is essential for maintaining biogenesis, and signals from this process are relayed to the nucleus via the NAD^+^/Sirt1/ERR transcriptional program, making spatial positioning information itself a regulatory component (62). Norepinephrine activates PGC-1α via the β_2_-adrenergic receptor cascade, directly coupling neurotransmitter signaling with mitochondrial renewal (63). Piezo1-mediated mechanotransduction promotes biogenesis through AMPK/SIRT1-dependent PGC-1α deacetylation, making mechanical force an additional upstream signal (64). Additionally, the activation of the AMPK-PGC-1α axis enhances mitochondrial biogenesis and triggers antibacterial xenophagy to restrict intracellular *M.tb* infection (65). Recent progress in this field thus comes less from identifying new regulatory factors than from revealing that mitochondrial biogenesis is a dynamic, cross-compartmental process that integrates spatial location, neurotransmitter, and mechanical signals. Its physiological logic can be fully understood only when biogenesis is placed within the full MQC framework and examined together with fusion and fission, mitophagy, and proteostasis.

### 2.5 Mitophagy: the terminal clearance mechanism

Mitophagy is the terminal, irreversible level of the MQC hierarchy. Unlike the reversible buffering strategies of tiers I–III, mitophagy delivers damaged mitochondria to lysosomes for degradation. It is an evolutionarily conserved, selective autophagy process that specifically recognizes, isolates, and clears dysfunctional or excess mitochondria, preventing them from releasing ROS, pro-apoptotic factors, and DAMPs that amplify cellular stress and inflammation (8, 26, 66, 67).

The PINK1-Parkin pathway is the most extensively studied damage-responsive mitophagy mechanism in mammalian cells (67, 68). In healthy mitochondria with intact ΔΨm, PINK1 is imported into the inner mitochondrial membrane, where mitochondrial processing peptidase (MPP) and the rhomboid protease PARL expose its N-terminal phenylalanine. PINK1 is then rapidly degraded via the N-end rule pathway and the ubiquitin-proteasome system, keeping PINK1 at low levels in healthy mitochondria (69). Depolarization or blocked protein import interrupts continuous PINK1 import and degradation. Instead, PINK1 accumulates stably on the outer mitochondrial membrane and is activated through autophosphorylation. Activated PINK1 phosphorylates Ser65 on pre-existing ubiquitin chains on the outer mitochondrial membrane to generate phosphorylated ubiquitin (pSer65-Ub), which provides a high-affinity binding site for Parkin, recruiting the otherwise autoinhibited cytoplasmic E3 ubiquitin ligase to the surface of damaged mitochondria and thereby activating it (70–72). Parkin then ubiquitinates various outer-membrane proteins, amplifying the “eat-me” signal on the surface of damaged mitochondria, and recruits autophagy receptors such as nuclear dot protein 52 (NDP52) and optineurin (OPTN). These receptors recognize pSer65-Ub chains on the mitochondrial surface through their ubiquitin-binding domains, and simultaneously bind LC3/GABARAP family proteins on the autophagosome membrane through their LIR (LC3-interacting region) motifs, anchoring the damaged mitochondria to the extending autophagosome membrane (73). At the same time, these receptors recruit autophagy initiation factors, such as the unc-51 like kinase 1 (ULK1) complex, double FYVE-containing protein 1 (DFCP1), and WD repeat domain phosphoinositide-interacting protein 1 (WIPI1), to focal regions surrounding the mitochondria, thereby initiating autophagosome nucleation and extension (74) (**Figure 1D**).

In addition to the classical mitophagy pathway mediated by PINK1-Parkin, a receptor-mediated pathway involving BNIP3, a member of the Bcl-2 family, and its homolog NIX is another important mitophagy pathway (75). The BNIP3 pathway primarily reflects the role of mitophagy in metabolic reprogramming during hypoxic adaptation. The transmembrane domains of these receptors anchor them stably to the outer membrane. Their cytoplasmic ends contain LIR motifs that bind directly to LC3/GABARAP proteins on the autophagosome membrane. Damaged or redundant mitochondria can thus be delivered to the autophagy system without relying on ubiquitination, enabling “receptor-mediated mitophagy.” This pathway is particularly critical in stress responses, hypoxic environments, and the development of erythroid cells (**Figure 1D**).

Localized to the outer mitochondrial membrane, FUNDC1 binds LC3 through its LIR domain under stress conditions such as hypoxia or ischemia-reperfusion, directly driving damaged mitochondria into the autophagy pathway (76). FUNDC1 participates in autophagy recognition and is also closely coupled to mitochondrial fission, integrating “mitochondrial sorting” and “mitochondrial clearance” into a continuous event (77) (**Figure 1D**).

To date, the molecular repertoire of receptor-dependent mitophagy has continued to expand. BCL2L13 has been proposed as a receptor-like molecule in mammals with a function similar to that of yeast Atg32, capable of simultaneously promoting mitochondrial fragmentation and autophagic clearance (78). FK506 binding protein 8 (FKBP8), by contrast, exhibits a preferential ability to recruit LC3A, suggesting that different ATG8 family members may selectively execute different receptor pathways (79). Activating molecule in beclin-1 regulated autophagy 1 (AMBRA1) can both promote classical Parkin-dependent mitochondrial clearance and, under specific conditions, mediate a relatively independent form of mitophagy through LC3 binding (80, 81). These studies collectively indicate that mammalian mitophagy functions more as a network built from multiple receptors and cofactors than as a single pathway.

Beyond whole-organelle mitophagy, mitochondria-derived vesicles (MDVs) may provide an alternative MQC route when mitophagic clearance is impaired. In *M.tb*-infected human macrophages, p62/SQSTM1 depletion impaired mitophagic flux but enhanced mitochondrial Rho GTPase 1/2 (MIRO1/MIRO2)-dependent TOM20^+^ MDV biogenesis. The resulting vesicles were recruited to *M.tb*-containing phagosomes, where they were associated with Ras-related protein Rab-7 (RAB7) recruitment, reversal of phagosome maturation arrest, and increased lysosomal targeting of the bacteria (82).

This continuous, four-tier spectrum of responses matches repair capacity to the extent of damage. Its highly integrated design also implies, however, that altering the intensity and timing of damage signals can reset the switching thresholds between tiers. *M.tb* interferes with host mitochondrial homeostasis precisely through this pathway. Before the specific manipulation of each MQC tier is analyzed, the mitochondrial damage caused by infection itself warrants examination first, including morphological and dynamic changes, metabolic reprogramming, and the three damage signaling axes of mtROS, mtDNA, and ΔΨm.

## 3 *M.tb* infection-induced mitochondrial homeostasis dysregulation

### 3.1 Mitochondrial morphology and dynamics

Numerous studies have documented marked changes in mitochondrial morphology caused by *M.tb* infection. In both *in vitro* and *in vivo* models, macrophages infected with the highly virulent *M.tb* strain H37Rv exhibit mitochondria that lose their characteristic rod-like morphology, becoming swollen, spherical, and structurally disorganized (83). This may be accompanied by disruption of cristae structures (84) and can lead to necrosis. By contrast, the attenuated strain H37Ra leads to apoptosis (85), while also inhibiting normal mitochondrial function and reducing ATP production (86) (**Table 1**).

**Table 1:** Mtb-induced mitochondrial damage.

Damage axis	Strain/antigen	Key molecular events and effectors	Functional consequence	Model system	Reference(s)
1. Structural disruption	H37Rv	Rod-shaped mitochondria become swollen, spherical and structurally disorganized; cristae disrupted; MPT induced and ΔΨm dissipated	Necrosis	*In vitro* (macrophages) + *in vivo* (mouse)	(83, 84, 108)
	H37Ra	Outer mitochondrial membrane permeabilized; ATP production reduced	Apoptosis	*In vitro* (macrophages) + *in vivo* (mouse)	(85, 108)
2. Fusion–fission imbalance	H37Rv (early phase)	MFN2 sustains mitochondrial function, promoting NLRP3 assembly/IL-1β secretion and HIF-1α-dependent aerobic glycolysis and xenophagy	Host-protective immune responses	Human PBMCs from active TB patients + macrophages *in vitro*; mouse BMDM *in vitro* + *in vivo*	(88, 89)
	H37Rv (middle–late phase)	ER stress upregulates Parkin; DRP1 and pMFF upregulated; MFN2 ubiquitinated and degraded, driving fragmentation. MFN2 knockdown limits Mtb survival, overexpression promotes proliferation; fusion defects cause growth retardation, ΔΨm loss and respiratory-chain decline	Pathogen-favoring fragmentation and macrophage apoptosis	Macrophages *in vitro*; MEFs *in vitro*	(90, 91)
	*M.tb*	MFN1-mediated mitochondrial fusion enhances OXPHOS and ATP production	Supports autophagic killing	Macrophages *in vitro*	(92)
	Rv1411c (LprG/P27)	Induces mitochondrial fission, decreases cellular respiratory rate, and alters mitochondrial Ca²^+^ uptake	Differential remodeling of mitochondrial dynamics and bioenergetics	Human macrophages *in vitro*	(87)
	Rv1818c (PE_PGRS33)	Induces mitochondrial fusion without appreciable changes in cellular respiration or mitochondrial Ca²^+^ uptake			
3. Bioenergetic failure	H37Rv vs H37Ra	H37Rv (but not H37Ra) significantly disrupts the inner mitochondrial membrane	Necrosis	Macrophages *in vitro*	(85, 108)
	TNT	Depletes NAD^+^, triggering RIPK3/MLKL necroptosis with ΔΨm loss and impaired ATP synthesis; GAPDH inhibited, depressing glycolytic flux	Loss of metabolic adaptability	THP-1 *in vitro*	(100–102)
4. Metabolic reprogramming	*M.tb*	4–8 h post-infection: M1 polarization with OXPHOS→glycolysis (Warburg); 24–48 h: M2 polarization with return to OXPHOS driven by exogenous fatty acids	Early defensive metabolism, late permissive state	Review of mouse data; macrophages *in vitro*	(93–99)
	Live virulent M.tb vs heat-killed M.tb/BCG	Live bacilli drive a nearly quiescent bioenergetic state; heat-killed Mtb/BCG activates glycolysis	Active reshaping of host metabolism	Human MDMs *in vitro*	(99, 103)
	*M.tb*	Serum and tissue succinate elevated; glyoxylate bypass and methylcitrate cycle activated	Metabolic adaptation within the granulomatous microenvironment	C57BL/6 mice *in vivo*	(103)
5. mtROS dysregulation	Rv0928 (effector)	Amplifies macrophage mtROS and inflammation	Strengthens host control of mycobacteria	Macrophages *in vitro*	(111)
	TNF (host cytokine)	Induces mtROS via complex I reverse electron transport; in excess activates BAX, Ca²^+^ overload and cyclophilin D-dependent necrosis	Bacterial release into the extracellular space, exacerbating infection	Zebrafish *in vivo*	(113–115)
	*M.tb*	Threshold-dependent mtROS: moderate levels support cytokines, phagocytosis, autophagy, apoptosis and bacterial control; excessive levels oxidize membrane lipids and drive pathological inflammation; mtROS activates NLRP3 and directs GSDMD to mitochondria	Pyroptosis→necroptosis switch	Mouse *in vivo*; BMDM/THP-1 + mouse *in vivo*; macrophages *in vitro*	(116, 117)
6. mtDNA release	*M.tb*	mtDNA escapes via mPTP/VDAC channels; cGAS sensing amplifies type I IFN through STING-IRF3 and further activates NLRP3	Pathogenic type I IFN signature	Mouse BMDM/MEFs + mouse *in vivo*; macrophages *in vitro*	(118–120)
	H37Rv vs other clinical isolates	H37Rv induces greater cytoplasmic mtDNA accumulation and cGAS-STING-dependent IFNβ; MitoQ reduces mtDNA release and IFNβ	Type I IFN signature associated with TB progression	Mouse BMDMs *in vitro*	(120)
7. Ferroptosis induction	*M.tb*	GSH depletion, GPX4 downregulation, free-iron accumulation and lipid peroxide production; Fer-1 blocks cell death and reduces bacterial load *in vivo*	Primary pathway of macrophage and tissue necrosis	Mouse *in vivo*; macrophages *in vitro*	(104)
	BACH1 (host transcription factor)	Represses the Nrf2 pathway, downregulating GPX4 and Gclm	Mitochondrial ROS accumulation and membrane lipid peroxidation	Human patient cohort; mouse *in vivo*	(105)
	PtpA (effector)	Enters the nucleus, binds PRMT6 and raises H3R2me2a to epigenetically silence GPX4	Synergistically promotes ferroptosis	Macrophages *in vitro*	(106)
	Rv1324 (effector)	Induces local ROS bursts, GSH depletion and GPX4 inhibition	Aggravates ferroptosis-dependent pulmonary necrosis and inflammation	Mouse *in vivo*	(107)
8. Integrated multi-axis attack	ESAT-6 (effector)	Drives GSDMD cleavage; GSDMD-NT translocates to mitochondria, simultaneously causing ΔΨm loss, mtDNA release and mtROS production	Synergistic cGAS-STING activation and amplified IFNβ, uniting all three damage axes into a single pathogenic program	Macrophages *in vitro*	(121)

In addition to passive structural damage, *M.tb* actively manipulates mitochondrial fusion and fission. Rv1411c (LprG/P27) induces mitochondrial fission, decreases the cellular respiratory rate, and alters mitochondrial Ca^2+^ uptake in human monocyte-derived macrophages, whereas Rv1818c (PE_PGRS33) promotes mitochondrial fusion without appreciably affecting cellular respiration or mitochondrial Ca^2+^ uptake (87). In the early stages of infection, macrophage MFN2 strengthens the anti-*M.tb* immune response by maintaining mitochondrial function, promoting NLR family pyrin domain containing 3 (NLRP3) inflammasome assembly and Interleukin (IL)-1β secretion (88), and facilitating hypoxia-Inducible Factor 1 subunit α (HIF-1α)-mediated aerobic glycolysis and xenophagy (89). The highly virulent strain H37Rv can upregulate the E3 ubiquitin ligase Parkin via endoplasmic reticulum stress. During the middle to late stages of infection, it upregulates the fission proteins dynamin-related Protein 1 (DRP1) and phosphorylated mitochondrial fission factor (pMFF) and mediates the ubiquitination and degradation of MFN2, inducing mitochondrial fragmentation and thereby promoting macrophage apoptosis. Artificial knockdown of MFN2 significantly inhibits the intracellular survival of *M.tb*, whereas its overexpression promotes bacterial proliferation, identifying MFN2 as a major regulator of *M.tb* intracellular survival (90). This fusion defect further leads to growth retardation, loss of ΔΨm, and complete decline in respiratory chain function (91). Meanwhile, Ning et al. (92) showed that during *M.tb* infection, macrophages can also induce mitochondrial fusion via MFN1, increasing oxidative phosphorylation and ATP production and thereby promoting autophagic killing. Through differential regulation of MFN1/MFN2-mediated fusion and fission, *M.tb* reshapes the macrophage immune response (**Figure 2A**; **Table 1**).

**Figure 2:**
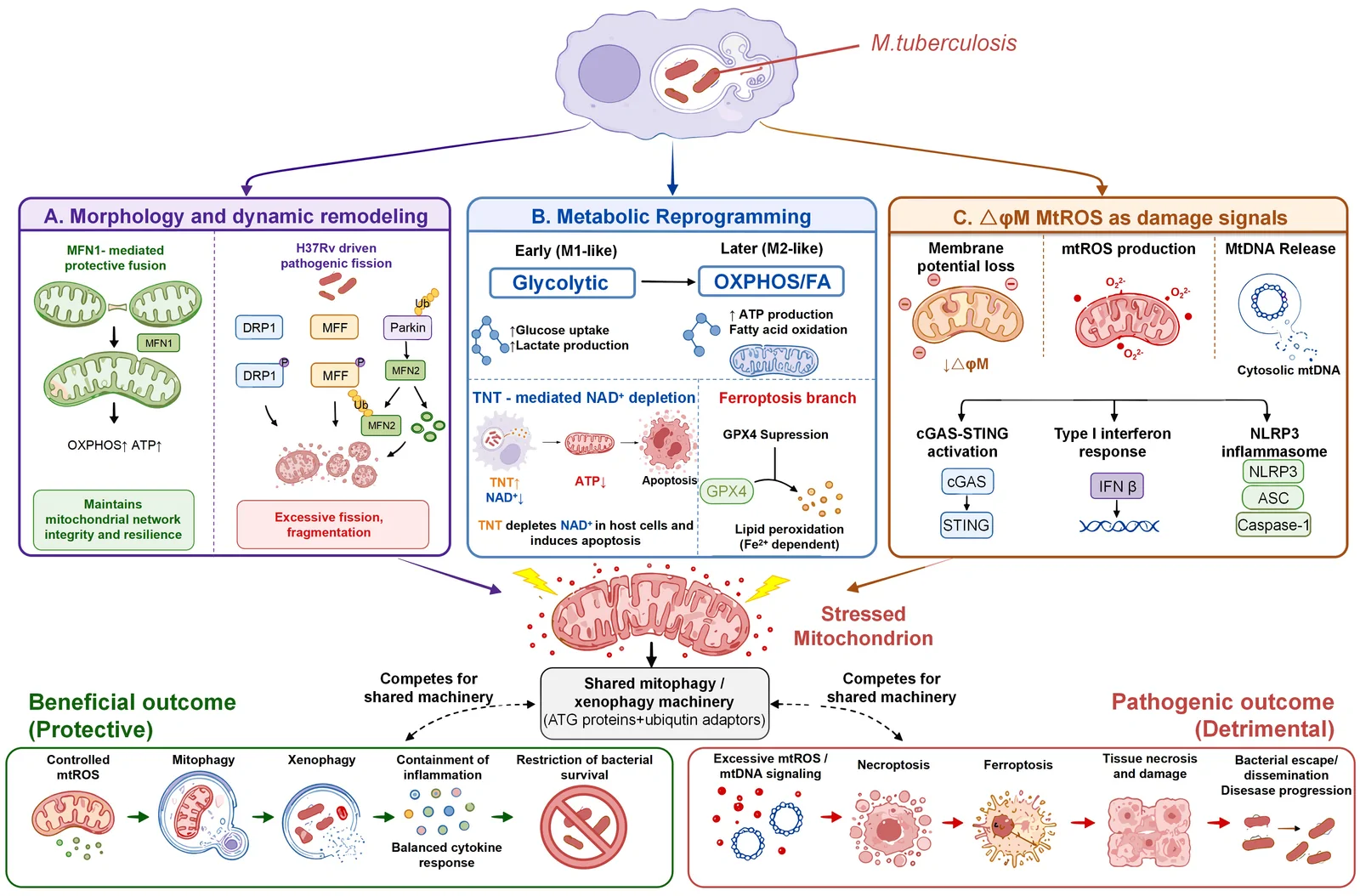
*M.tb*-induced mitochondrial remodeling and damage signaling shape divergent host outcomes. *M.tb* disrupts mitochondrial homeostasis through coordinated alterations in mitochondrial dynamics, metabolic reprogramming, and mitochondrial damage signaling. **(A)** Morphology and dynamic remodeling. MFN1-mediated mitochondrial fusion supports network integrity, oxidative phosphorylation, and ATP production, whereas virulent H37Rv promotes fragmentation through DRP1/MFF activation and Parkin-associated MFN2 ubiquitination and degradation. **(B)** Metabolic reprogramming. Early infection favors a glycolytic, M1-like state; later stages shift toward oxidative phosphorylation and fatty-acid utilization. The effector TNT)also depletes intracellular NAD^+^ and impairs bioenergetics, and GPX4 suppression promotes lipid peroxidation and ferroptotic injury. **(C)** Mitochondrial damage signaling. Loss of ΔΨm, accumulation of mtROS, and release of mtDNA activate interconnected innate immune pathways, including cGAS-STING signaling, type I interferon responses, and NLRP3 inflammasome activation. These processes converge on a stressed mitochondrial state in which mitophagy and antibacterial xenophagy may compete for shared autophagy machinery. The lower branches illustrate the context-dependent consequences of this stress: controlled mtROS production, mitochondrial quality control, xenophagy, and balanced inflammatory responses can contribute to bacterial restriction, whereas excessive or persistent mtROS/mtDNA signaling can promote necroptosis, ferroptosis, tissue injury, bacterial escape, and disease progression. MFN1/2, mitofusin 1/2; DRP1, dynamin-related protein 1; MFF, mitochondrial fission factor; OXPHOS, oxidative phosphorylation; FA, fatty acid oxidation; TNT, tuberculosis necrotizing toxin; NAD^+^, nicotinamide adenine dinucleotide; GPX4, glutathione peroxidase 4; ΔΨm, mitochondrial membrane potential; mtROS, mitochondrial reactive oxygen species; mtDNA, mitochondrial DNA; cGAS, cyclic GMP–AMP synthase; STING, stimulator of interferon genes; IFN, interferon; NLRP3, NLR family pyrin domain-containing 3; ASC, apoptosis-associated speck-like protein containing a CARD; ATG, autophagy-related protein.

### 3.2 Host metabolic reprogramming and bioenergetic imbalance

*M.tb* infection triggers biphasic mitochondrial metabolic reprogramming in macrophages (93). In the early stages of infection, macrophages adopt an M1-polarized state, accompanied by a shift in mitochondrial metabolism from OXPHOS to glycolysis (94–96). By the adaptation/remission phase, the cells transition to an M2-polarized state (97), and macrophage metabolism shifts back from glycolysis to mitochondrial OXPHOS driven by exogenous fatty acids (98, 99). This biphasic pattern establishes a temporal framework that is critical for understanding how *M.tb* disrupts host metabolism. The pathogen must survive the hostile early glycolytic environment while simultaneously steering macrophages toward a more permissive M2-like metabolic state (**Table 1**).

Recent studies have linked this metabolic remodeling to NAD^+^ homeostasis. *M.tb* depletes NAD^+^ within host cells by secreting tuberculosis necrotizing toxin (TNT), triggering RIPK3/MLKL-mediated necroptosis, accompanied by loss of ΔΨm and impaired ATP synthesis (100). At the same time, NAD^+^ depletion inhibits glyceraldehyde-3-phosphate dehydrogenase (GAPDH), a central enzyme of glycolysis, decreasing glycolytic flux and further impairing the metabolic adaptability of macrophages (101). Supplementing the NAD^+^ precursor nicotinamide restores macrophage glycolytic capacity via the NAD^+^ salvage pathway and significantly reduces the survival of *M.tb* within host cells. Both supplementation with glucose and knockdown of Akt inhibit glycolysis, thereby promoting the survival of *M.tb* within macrophages (**Figure 2B**) (102).

The metabolic consequences of *M.tb* infection differ markedly between highly virulent live bacteria and inactivated or attenuated strains. Live, highly virulent *M.tb* drives human macrophages into a nearly “quiescent” bioenergetic state, slowing glycolysis and the TCA cycle. In contrast, heat-inactivated *M.tb* or BCG tends to induce glycolytic activation (99). Shin et al. (103) found markedly elevated levels of succinate, a TCA cycle intermediate, in multiple organs and serum, together with decreased levels of oxalate and fumarate. This abnormality is closely associated with activation of the glyoxylate bypass and the methylcitrate cycle, oxidative stress, and host cell metabolic reprogramming, and it reflects the metabolic adaptations between pathogen and host within the granulomatous microenvironment.

Another important consequence of *M.tb*-induced metabolic disruption is lipid peroxidation-driven ferroptosis, characterized by glutathione (GSH) depletion, downregulation of glutathione peroxidase 4 (GPX4), accumulation of free iron, and massive production of toxic lipid peroxides. Extensive evidence, including the seminal study by Amaral et al. (104), has established ferroptosis as the primary pathway of *M.tb*-induced macrophage necrosis and tissue necrosis. At the molecular level, the host transcription factor BTB and CNC homology 1 (BACH1) downregulates GPX4 and main enzymes of glutathione synthesis by inhibiting the Nrf2 antioxidant pathway, leading to the accumulation of mitochondria-derived reactive oxygen species and membrane lipid peroxidation (105). In parallel, the *M.tb*-secreted effector protein PtpA enters the macrophage nucleus, binds to protein arginine methyltransferase 6 (PRMT6), and increases H3R2me2a modification, epigenetically silencing GPX4 transcription and thereby synergistically promoting ferroptosis (106). Furthermore, by secreting another virulence protein, Rv1324, *M.tb* directly induces local ROS bursts, GSH depletion, and inhibition of GPX4 activity, exacerbating ferroptosis-dependent pulmonary necrosis and inflammation (107). Inhibitory regulation by host BACH1 and active intervention by the bacterial effector proteins PtpA and Rv1324 thereby impair mitochondrial redox buffering capacity, driving infected cells toward necrosis and ultimately exacerbating tissue destruction and bacterial dissemination. Treatment with the specific ferroptosis inhibitor Fer-1 blocks cell death induced by these two pathways and significantly reduces bacterial load *in vivo* (105, 106). Host BACH1-mediated transcriptional regulation and multiple bacterial effector proteins converge on GPX4 inhibition and ferroptosis. This convergence marks the pathway as a critical node of host-pathogen interaction.

### 3.3 MtROS, mtDNA, and ΔΨm as damage signals

Loss of ΔΨm is a hallmark of mitochondrial dysfunction. It directly triggers apoptosis or necrosis. Both the virulent *M.tb* strain H37Rv and the attenuated strain H37Ra reduce ΔΨm, but only H37Rv drives the significantly opening of the mitochondrial permeability-transition (MPT) pore, irreversibly disrupting the inner mitochondrial membrane and accelerating necrotic macrophage death, which favors bacterial egress and dissemination. While H37Ra also dissipates ΔΨm, it does so only partially and promotes apoptosis rather than necrosis under standard low-MOI conditions (**Figure 2C**) (85, 108).

*M.tb* infection induces cells to produce large amounts of mtROS. As a signal of oxidative damage, mtROS activates host pro-inflammatory and apoptotic pathways and is a critical defensive signal in the host response against *M.tb* (109). When mitochondrial homeostasis is maintained, moderate and controlled levels of mtROS support cytokine production, phagocytosis and autophagy, apoptosis, and bacterial control (2, 102, 110). mtROS therefore acts as a threshold-dependent immunoregulator. Rv0928 can interact with host mitochondria-associated proteins, amplifying macrophage mtROS and associated inflammatory responses and ultimately strengthening the host’s control over mycobacterial infection (111). However, when this homeostatic balance is disrupted, excessive mtROS production damages mitochondrial components, oxidizes membrane lipids, and drives pathological inflammation (112). Roca et al. (113) demonstrated that TNF induces mtROS production in infected macrophages via reverse electron transport (RET) through mitochondrial complex I. TNF-induced mtROS initially increases macrophage bactericidal activity and limits infection. Once the TNF-mtROS axis becomes excessive, it rapidly triggers a mitochondrial-lysosomal-endoplasmic reticulum cascade, leading to BCL2-Associated X protein (BAX) activation, mitochondrial Ca²^+^ overload, and cyclophilin D-dependent necrosis (114), resulting in release of the bacteria into the extracellular space and exacerbation of infection (**Figure 2C**) (115).

Mitochondrial damage and mtROS are also important upstream signals for NLRP3 activation (116). When the inflammasome is activated, elevated mtROS directs gasdermin D (GSDMD) to act on the mitochondrial membrane, shifting cell death from pyroptosis toward necroptosis (117). This mtROS-dependent shift in the mode of cell death illustrates how mitochondrial damage signals can qualitatively alter the immunological consequences of cell death. Pyroptosis is typically pro-inflammatory and immunostimulatory, whereas necroptosis promotes bacterial dissemination (**Table 1**).

Mitochondrial DNA (mtDNA) is the third major damage signaling axis. *M.tb*-induced mitochondrial permeability transition (MPT) triggers mtDNA release, which acts as a DAMP to transmit signals of mitochondrial damage (85). As an endogenous DNA ligand recognized by the innate immune system, mtDNA escapes into the cytoplasm via the mPTP/VDAC channel, where it is recognized by cGAS and amplifies the type I interferon response through the STING-IRF3 pathway (118), further activating the NLRP3 inflammasome (119). Among *M.tb* strains, H37Rv induces greater cytoplasmic mtDNA accumulation and cGAS-STING-dependent IFNβ production than other clinical isolates (120). The mitochondria-specific antioxidant MitoQ reduces mtDNA release and partially inhibits IFNβ induction, confirming that mtDNA release is causally linked to the type I IFN response rather than merely associated with mitochondrial stress. Importantly, excessive type I IFN signaling is associated with TB disease progression in both animal models and human patients, making the mtDNA-cGAS-STING pathway a pathogenic signaling axis (**Figure 2C**) (120).

A recent study by Yang et al. (121) provides a compelling example of how a single *M.tb* virulence factor can synergistically activate all three damage signaling axes. The major virulence protein ESAT-6 induces GSDMD cleavage, and the resulting GSDMD-NT fragment translocates to mitochondria, disrupting mitochondrial integrity while simultaneously causing loss of ΔΨm, mtDNA release, and mtROS production. This synergistic damage activates the cGAS-STING pathway and amplifies the IFNβ response. 6 kDa early secretory antigenic target (ESAT-6) thus shows how a single mycobacterial effector protein can simultaneously trigger all three mitochondrial damage signaling axes, transforming seemingly independent damage pathways into an integrated pathogenic signaling program (**Figure 2C**).

The mitochondrial stress induced by *M.tb* is intense and persistent, often exceeding the compensatory capacity of the reversible tiers, thereby pushing the fate decision toward the terminal tier of mitophagy. This tier plays a dual role in *M.tb* infection. It irreversibly determines the clearance efficiency of damage signals such as mtROS and mtDNA, while simultaneously sharing the ATG machinery and ubiquitin-conjugating signaling with antimicrobial xenophagy, making it a node of competition between host and pathogen. This overlap is the root cause of the conflicting conclusions regarding its role in the existing literature. The following section argues that the outcome of mitophagy is set by the specific receptor pathway that is activated, not by whether the process occurs.

## 4 Pathway-specific outcomes of mitophagy in tuberculosis

### 4.1 The PINK1-Parkin axis: protective xenophagy and exploited mitophagy

The PINK1-Parkin pathway plays a dual role in mycobacterial infection, which illustrates the context-dependent nature of mitophagy outcomes. On the protective side, Parkin (Park2) is a central mediator of antibacterial xenophagy. Manzanillo et al. (122) demonstrated that Parkin is recruited to *M.tb*-containing phagosomes in an ESX-1 secretory system-dependent manner. On the phagosomal membrane perforated by *M.tb*, Parkin catalyzes the assembly of K63-linked polyubiquitin chains via its E3 ubiquitin ligase activity, which further recruits autophagy adaptor proteins including p62, NDP52 and neighbor of BRCA1 gene 1 (NBR1) and promotes the phosphorylation and activation of TBK1. These adaptor molecules subsequently recruit core autophagy components such as LC3 and ATG12, driving the nucleation and assembly of autophagosomes around the *M.tb*-containing phagosomal membrane. Ultimately, intracellular pathogens are transported to lysosomes for degradation. The E3 ubiquitin ligase activity of Parkin serves as an essential prerequisite for this cascade reaction. Knockout of the *Park2* gene markedly impairs *M.tb* ubiquitination modification, autophagic targeting and lysosomal clearance, leading to massive proliferation of intracellular *M.tb*. *In vivo* infection models also verify that *Park2^−^/^−^* mice are highly susceptible to *M.tb*, with significantly increased bacterial burdens in multiple tissues and organs, and eventually die from *M.tb* infection. This fully demonstrates that Parkin is a key core molecule for hosts to resist the invasion of *M.tb* through the ubiquitin-autophagy pathway (**Table 2**).

**Table 2:** Pathway-specific outcomes and pharmacological regulation of mitophagy in TB.

Pathway/receptor	Trigger/intervention	Key molecular events and effectors	Outcome for infection	Model system	Reference(s)
PINK1-Parkin axis	*M.tb*	Parkin recruited to M.tb-containing phagosomes; catalyzes K63-linked ubiquitin chains, recruits p62/NDP52/NBR1 and phosphorylates TBK1, driving antibacterial xenophagy and lysosomal clearance	Host-protective	Macrophages *in vitro* + mouse *in vivo*	(122)
	*M. bovis*	Activates PINK1-Parkin mitochondrial autophagy, competitively depleting p-TBK1 and suppressing host xenophagy	Pro-pathogen (immune evasion and intracellular survival)	Macrophages *in vitro*	(13)
	ESAT-6 (bacterial effector)	Disrupts the phagosome membrane, releasing HK2 from VDAC; VDAC oligomerizes and forms outer-membrane pores, causing mitochondrial damage and activating mitophagy	Pro-pathogen (mitophagy hijacked by M. bovis)	Macrophages *in vitro*	(124)
	HABA (bacterial effector)	suppressing mitochondria–lysosome formation and PINK1/Parkin-associated mitophagy	Pathogen-favoring mitochondrial injury	Macrophages *in vitro*	(125)
BNIP3	*M.tb*	mtROS activates HIF-1α, upregulating BNIP3; mitophagy clears damaged mitochondria and lowers mtROS and pro-inflammatory TNF-α/MCP-1	Pro-pathogen (permissive intracellular environment); BNIP3 knockout/knockdown reverses inflammation and suppresses M.tb survival	Macrophages *in vitro* + mouse *in vivo*	(14)
NIX	rD4GDI (host factor from apoptotic Tregs)	Upregulates the mitophagy receptor NIX, promoting mitophagy and antimicrobial peptide PLA2G7 expression	Host-protective (anti-M.tb via mtROS-mediated IL-1β)	Human macrophages *in vitro*	(126)
	*M.tb*	NIX-mediated mitophagy reprograms cellular metabolism	Host-protective (M1 polarization and pro-inflammatory secretion, supporting immune clearance)	Phagocytic cells *in vitro*	(127)
FUNDC1	BNIP3-deficient background	PINK1 and FUNDC1 upregulated as compensation; further FUNDC1 knockdown aggravates mitophagy defects, raises mtROS and pro-inflammatory factors, and reduces M.tb survival	Pathogen-supportive compensation in BNIP3-deficient macrophages	Cell lines *in vitro*	(14)
Pharmacological regulation (drugs)	Thiopeptide antibiotics	Specifically induce mitophagy, independent of antibacterial and proteasome-inhibiting activities	Neutral (antibacterial activity occurs independently of mitophagy)	Cell lines *in vitro*	(132)
	Mitoxantrone (MTX)	Induces ATP depletion, ΔΨm depolarization and mtROS elevation, triggering mitochondrial fission and PINK1-Parkin mitophagy to clear dysfunctional mitochondria	Host-protective (synergizes with autophagy to reduce intracellular mycobacterial survival)	Human macrophages *in vitro*	(128)
	SMIP-30/SMIP-031 (PPM1A inhibitors)	Increase S403-p62 phosphorylation and activate selective autophagy	Host-protective (limit M.tb survival in macrophages and mouse lungs; reduce splenic bacterial load in infected mice)	Macrophages *in vitro* + mouse *in vivo*	(129)
	Imiquimod (TLR7 agonist)	Early phase: raises mtROS via p38 and MEK/ERK1/2, upregulates BNIP3 and binds Beclin-1, targeting autophagosomes to mitochondria; late phase: induces NO via GSK-3β	Host-protective (dose-dependent inhibition of intracellular H37Rv growth)	Mouse macrophages *in vitro*	(130, 131)

However, the PINK1-Parkin mechanism can also be co-opted to work against the host. Song et al. (13) revealed that *M. bovis* activates PINK1-Parkin-mediated mitophagy in macrophages, and that this mitophagy paradoxically promotes bacterial immune evasion. mitophagy inhibits host xenophagy by competitively depleting phosphorylated TBK1 (p-TBK1), thereby helping *M. bovis* evade macrophage-mediated killing and promoting its intracellular survival. Adriaenssens et al. (123) recently provided the molecular basis for this TBK1 competition mechanism from a structural biology perspective. NAK-associated protein 1 (NAP1) and similar to NAP1 TBK1 adaptor (SINTBAD) raise the threshold for the initiation of mitophagy by competing with OPTN for TBK1 binding, while simultaneously stabilizing NDP52-FAK family kinase-interacting protein of 200 kDa (FIP200) to drive the autophagy process. Li et al. (124) described a second, mechanistically independent pathway, in which ESAT-6 from *M. bovis* disrupts the phagosome membrane, triggering the release of hexokinase 2 (HK2) from its normal binding to the mitochondrial VDAC channel. HK2 dissociation induces VDAC oligomerization and the formation of pores in the outer mitochondrial membrane, leading to mitochondrial damage and the activation of mitophagy. Notably, this mitophagy, rather than limiting infection, is hijacked by *M. bovis* to promote its intracellular survival. The PINK1-Parkin pathway can thus drive opposing outcomes in mycobacterial infection. Parkin-mediated xenophagy protects the host by degrading bacteria, whereas excessive PINK1-Parkin-mediated mitophagy can be exploited by the pathogen to suppress xenophagy or expand its intracellular niche.

More recently, Heparin-binding hemagglutinin adhesin (HBHA) has been reported to suppress PINK1/Parkin-associated mitophagy and mitochondria-lysosome formation in macrophages (125). Mycobacterial proteins may therefore regulate this pathway by activating mitophagy, exploiting it, or inhibiting mitochondrial clearance.

### 4.2 Bnip3/NIX: context-dependent pro- and anti-bacterial functions

The BNIP3/NIX receptor-mediated mitophagy pathway further illustrates the principle of pathway-specific outcomes in TB. Lee et al. (14) revealed that BNIP3-mediated mitophagy is exploited by *M.tb* to create a permissive intracellular environment. *M.tb* infection induces mitochondrial damage and mtROS production, which in turn activates the HIF-1α pathway to upregulate BNIP3 expression. Acting as the principal receptor, BNIP3 mediates mitophagy to clear damaged mitochondria, reduce mtROS levels, and simultaneously decrease the release of pro-inflammatory factors such as TNF-α and MCP-1, thereby creating favorable conditions for the intracellular survival of *M.tb*. Knocking out or knocking down BNIP3 blocks *M.tb*-induced aberrant mitophagy, leading to massive accumulation of damaged mitochondria and a significant increase in mtROS, which in turn strengthens the pro-inflammatory immune response. This effectively suppresses *M.tb* intracellular survival and pulmonary bacterial load, both in macrophages *in vitro* and in mice. These findings identify BNIP3 as a potential therapeutic target. Pharmacological inhibition of BNIP3 restores inflammatory mtROS signaling and strengthens the anti-TB immune response (**Table 2**).

Unlike BNIP3, BNIP3L/NIX can mediate protective anti-mycobacterial effects. Rho GDP dissociation inhibitor D4GDI (rD4GDI), produced by regulatory T cells (Tregs), promotes the accumulation of LC3B in mitochondria and activates mitophagy in *M.tb*-infected human macrophages by upregulating the mitophagy receptor NIX. This induces expression of the antimicrobial peptide PLA2G7, which subsequently exerts an anti-*M.tb*- mtROS-mediated IL-1β production (126). At the same time, *M.tb* can also reprogram cellular metabolism through NIX-mediated mitophagy, driving macrophage polarization toward the pro-inflammatory M1 phenotype and secretion of pro-inflammatory factors, thereby regulating immunometabolism and contributing to the immune clearance of *M.tb* infection (127) (**Table 2**).

A comparison of BNIP3 and NIX is illuminating. These two closely related mitophagy receptors, both regulated by HIF-1α under stress conditions, mediate opposite effects on the outcome of infection. BNIP3 is exploited by *M.tb* to suppress inflammation, whereas NIX drives protective inflammatory polarization. This intra-family divergence strongly supports the framework of pathway specificity.

### 4.3 FUNDC1: compensatory redundancy in receptor-mediated mitophagy

Research on FUNDC1 during *M.tb* infection remains limited, and most of the evidence comes from BNIP3-deficient macrophages. Lee et al. (14) found that PINK1 and FUNDC1 were upregulated in these cells after infection, consistent with compensatory activation of alternative mitophagy pathways. Knockdown of FUNDC1 then reduced mitophagy, raised mitochondrial ROS and pro-inflammatory cytokine production, and lowered intracellular M.tb survival. Under BNIP3 deficiency, this compensatory mitophagy appears to favor bacterial persistence by restraining antimicrobial mitochondrial ROS and inflammatory signaling. This inference is limited by the model used. The experiments were performed in BNIP3-deficient murine macrophages, and whether FUNDC1 favors the bacteria or the host in primary human macrophages, or in human infection *in vivo*, remains undetermined (**Table 2**).

Overall, these findings support a stage- and pathway-dependent model of mitophagy during mycobacterial infection (**Figure 3**). Early infection shows M1-like glycolysis, controlled mtROS production, preserved ΔΨm, and xenophagy-associated bacterial restriction. During the intermediate phase, metabolic reprogramming toward OXPHOS/FA, HIF-1α-BNIP3 activation, NAD^+^ depletion, HK2-VDAC disruption, and competition for p-TBK1 create a tipping point between antibacterial xenophagy and mitophagy. At the chronic granuloma stage, excessive mtROS, ΔΨm collapse, and mtDNA release can amplify cGAS-STING signaling and promote ferroptosis, necrosis, tissue injury, and dissemination. Across these stages, PINK1-Parkin, BNIP3, NIX/BNIP3L, and FUNDC1 each retain distinct, context-dependent effects on host protection and bacterial persistence.

**Figure 3:**
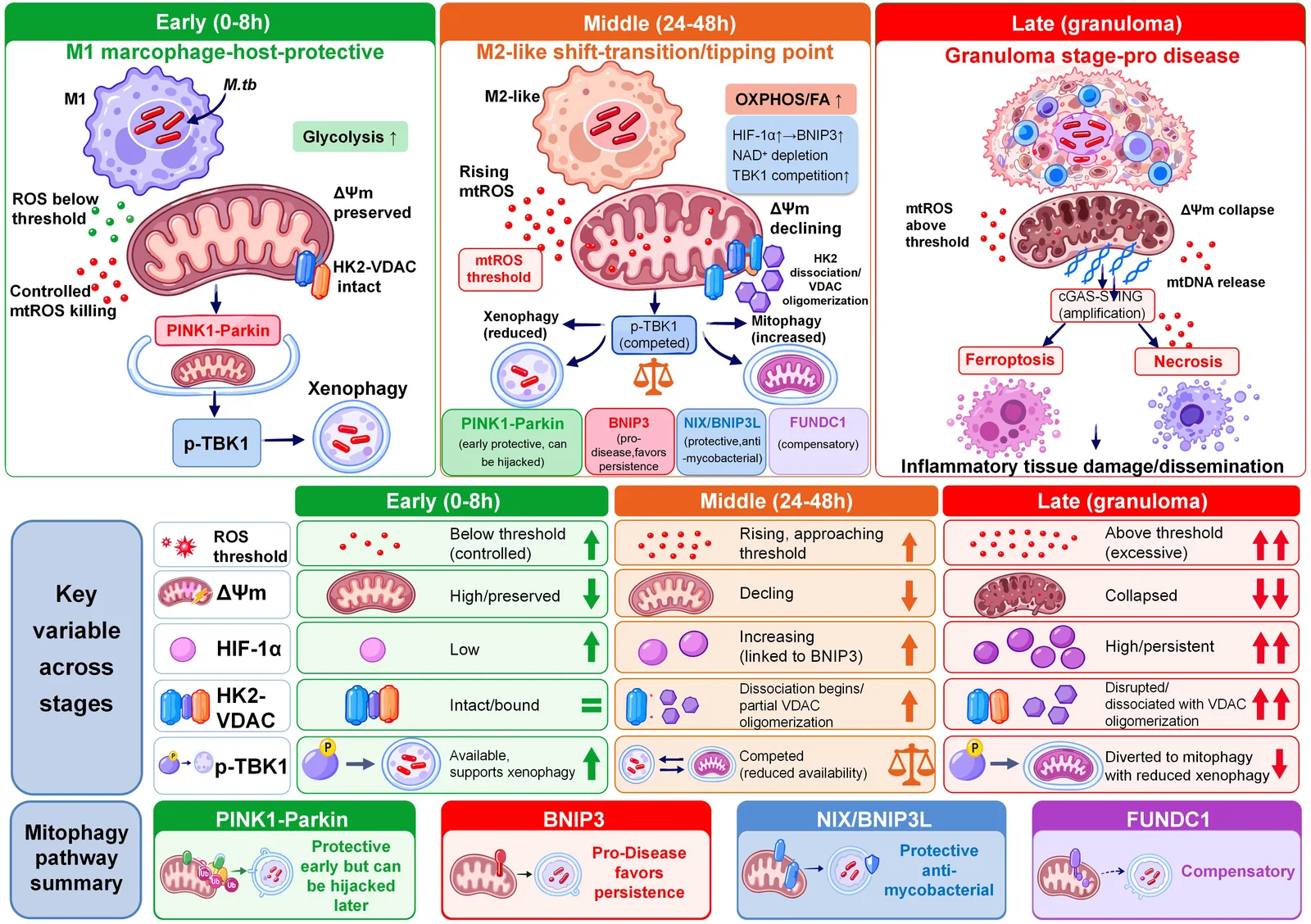
Stage-dependent decision model of mitophagy during *M.tb* infection. This model integrates the temporal evolution of macrophage immunometabolism, mitochondrial damage, and pathway-specific mitophagy during M. tuberculosis infection. During the early stage (0–8 h), macrophages adopt an M1-like, glycolysis-dominant state with controlled mtROS production, preserved ΔΨm, and an intact HK2-VDAC complex. Under these conditions, PINK1-Parkin-dependent quality control and p-TBK1-supported xenophagy restrict bacterial growth. During the middle stage (24–48 h), macrophages progressively shift toward an M2-like metabolic state with increased oxidative phosphorylation and fatty-acid oxidation. Rising mitochondrial stress approaches a functional ROS threshold, with declining ΔΨm, HIF-1α-dependent BNIP3 induction, TNT-associated NAD^+^ depletion, and HK2 dissociation and VDAC oligomerization. Competition for p-TBK1 and shared autophagy machinery favors mitophagy at the expense of antibacterial xenophagy, creating a tipping-point state. Individual mitophagy pathways diverge: PINK1-Parkin can be host-protective but may also be hijacked by mycobacteria; BNIP3-mediated mitophagy favors bacterial persistence in the models examined; NIX/BNIP3L-associated mitophagy supports anti-mycobacterial responses; and FUNDC1 provides compensatory, context-dependent mitophagy. During the late granuloma stage, persistent mitochondrial injury is associated with ΔΨm collapse, mtROS accumulation above the protective threshold, and mtDNA release through mPTP/VDAC-associated pathways. Cytosolic mtDNA activates the cGAS-STING axis and amplifies inflammatory signaling. Together with ferroptosis and necrosis, these events promote tissue injury and may facilitate bacterial dissemination. The lower panels summarize the stage-dependent behavior of the key decision variables (ROS threshold, ΔΨm, HIF-1α, HK2-VDAC integrity, and p-TBK1 availability) and the divergent effects of the mitophagy pathways. The indicated temporal windows represent a conceptual synthesis across different experimental systems rather than a single experimentally resolved time course. M1, classically activated macrophage phenotype; M2, alternatively activated macrophage-like phenotype; mtROS, mitochondrial reactive oxygen species; ΔΨm, mitochondrial membrane potential; OXPHOS, oxidative phosphorylation; FA, fatty-acid oxidation; HIF-1α, hypoxia-inducible factor 1α; BNIP3, BCL2-interacting protein 3; NIX/BNIP3L, BCL2-interacting protein 3-like; NAD^+^, nicotinamide adenine dinucleotide; TBK1, TANK-binding kinase 1; p-TBK1, phosphorylated TBK1; HK2, hexokinase 2; VDAC, voltage-dependent anion channel; PINK1, PTEN-induced kinase 1; FUNDC1, FUN14 domain-containing protein 1; mtDNA, mitochondrial DNA; mPTP, mitochondrial permeability transition pore; cGAS, cyclic GMP–AMP synthase; STING, stimulator of interferon genes; Ub, ubiquitin.

### 4.4 Pharmacological regulation of mitophagy in TB

Several recent studies have demonstrated the pharmacological accessibility of the mitophagy pathway, although all evidence remains at the preclinical stage. When mitoxantrone (MTX) acts on human macrophages at non-cytotoxic doses, it induces mitochondrial fission and activates PINK1-Parkin pathway-mediated mitophagy to clear dysfunctional mitochondria by triggering ATP depletion, ΔΨm depolarization, and a specific increase in mtROS. This mitophagy acts synergistically with autophagy to significantly reduce the survival of mycobacteria within macrophages (128). Regarding autophagy-activating host-directed therapy (HDT), the PPM1A-selective inhibitor SMIP-30 developed by Berton et al. (129) activates selective autophagy by increasing S403-p62 phosphorylation, thereby limiting the survival of *M.tb* in macrophages and mouse lungs. Yu et al. (130) further optimized the compound to obtain SMIP-031, which significantly reduced bacterial load in the spleens of *M.tb*-infected mice. Imiquimod (IMQ), a TLR7 agonist, initially increases mtROS via the p38 and MEK/ERK1/2 pathways, upregulates BNIP3, and promotes its binding to Beclin-1, directing autophagosomes toward mitochondria to initiate mitophagy. In later stages, it further promotes autophagy by inducing NO production via the GSK-3β pathway, thereby dose-dependently inhibiting the intracellular growth of H37Rv (131) (**Table 2**).

Bird et al. (132) showed that thiopeptides induce selective mitophagy independently of their intrinsic antibacterial and proteasome-inhibitory activities. Although several natural thiopeptides reduce intracellular Mtb burden in macrophage infection models, the mitophagy they induce occurs outside Mtb-containing autophagosomes and does not directly contribute to bacterial clearance. The two activities are therefore mechanistically independent, and mitophagy is not the pathway through which thiopeptides act on *M.tb*.

Mitophagy exhibits highly context-dependent functional differentiation during *M.tb* infection. The PINK1-Parkin pathway can mediate protective xenophagy or be hijacked by the pathogen. BNIP3 and NIX drive pro-bacterial and anti-bacterial effects, respectively, and FUNDC1 provides compensatory redundancy. The core challenge in pharmacological regulation is achieving pathway selectivity rather than merely demonstrating that mitophagy can be modified. Signal axes hijacked by pathogens must be avoided, while protective pathways should be strengthened. Such strategies must also consider that *M.tb* can bidirectionally manipulate mitophagy through different effector proteins. A central direction for the next phase of research is to systematically elucidate the hierarchical relationships among different receptors during infection and to develop receptor-selective strategies for regulating mitophagy.

## 5 Perspective

The multilevel MQC framework proposed here is an organizational model rather than a universally established linear hierarchy during *M.tb* infection. The studies integrated here span substantially different experimental systems, including primary human macrophages, THP-1 cells, RAW264.7 macrophages, murine bone-marrow-derived macrophages, PBMC-derived populations, mouse models, and zebrafish models. Mycobacterial species and strains also differ, spanning *M.tb* H37Rv, H37Ra, *M. bovis*, and BCG. Infection dose, multiplicity of infection, sampling time, metabolic conditions, and experimental endpoints vary as well. A pathway that appears protective in one setting should therefore not be assumed to have the same effect in another.

Direct evidence is also uneven across tiers of MQC. Mycobacterial effectors have been linked to chaperones, protein-import machinery, mitochondrial dynamics, redox regulation, and mitophagy-associated pathways, but effector-host target relationships have been established for only a few of these nodes (**Table 3**). *M.tb* chaperonin Cpn60.2 (GroEL2) directly interacts with mt-HSP70 (133), and *M.tb*-induced LINC02528, a host lncRNA, regulates TOMM22 (134). Conversely, no defined mycobacterial effector has been shown to target canonical upstream MQC components directly, including LONP1/ClpP, Hsp60/Hsp10, TIM23, OMA1/YME1L, the DELE1-HRI-eIF2α-ATF4 axis, and the PGC-1α-NRF-TFAM pathway. Thus, downstream phenotypes such as ΔΨm loss, mtROS accumulation, or mtDNA release should not by themselves be interpreted as evidence that an upstream MQC node is directly targeted.

**Table 3:** *M.tb* effector-MQC node evidence matrix.

Effector/axis	Direct host target/established mechanism	Proteostasis/chaperones	TOM/TIM	OMA1/YME1L	DELE1-HRI-eIF2α-ATF4	PGC-1α-NRF-TFAM	Dynamics	Mitophagy	MDV	ΔΨm/mtROS/mtDNA	GPX4/ferroptosis	Reference(s)
Cpn60.2/GroEL2	Directly binds mortalin/mtHsp70	Known direct	Indirect relevance	Unknown	Unknown	Unknown	Unknown	Unknown	Unknown	Indirect	Unknown	(133)
LINC02528	Directly binds TOMM22	Unknown	Known direct	Unknown	Unknown	Unknown	Functional	Unknown	Unknown	Known functional	Unknown	(134)
HBHA	Localizes to mitochondria; a 2026 study shows inhibition of PINK1/Parkin and mitochondria-lysosome fusion	Unknown	Unknown	Unknown	Unknown	Unknown	Indirect	Known functional	Unknown	Known direct/functional	Unknown	(125)
ESAT-6	GSDMD-NT mitochondrial translocation; HK2-VDAC mechanism (*M. bovis*)	Unknown	Unknown	Unknown	Unknown	Unknown	Indirect	Functional/indirect	Unknown	Known direct	Unknown	(121)
PtpA	PRMT6-H3R2me2a → GPX4 inhibition	Unknown	Unknown	Unknown	Unknown	Unknown	Unknown	Indirect inference	Unknown	Indirect	Known direct	(106)
Rv1324	Antioxidant/redox function; promotes ferroptotic lung injury	Unknown	Unknown	Unknown	Unknown	Unknown	Unknown	Indirect inference	Unknown	Known functional	Known functional	(107)
TNT	NAD^+^ glycohydrolase → NAD^+^ depletion → RIPK3/MLKL	Unknown	Unknown	Unknown	Unknown	Unknown	Unknown	Indirect inference	Unknown	Known functional	Unknown	(100–102)
LprG/P27	Induces mitochondrial fission and reduces respiration	Unknown	Unknown	Unknown	Unknown	Unknown	Known functional	Unknown	Unknown	Known functional	Unknown	(87)
PE_PGRS33	Alters mitochondrial dynamics and metabolism	Unknown	Unknown	Unknown	Unknown	Unknown	Known functional	Unknown	Unknown	Known functional	Unknown	
*M.tb* -MFN2/Parkin axis	ER stress → Parkin ↑ → MFN2 degradation; DRP1/pMFF ↑	Unknown	Unknown	Unknown	Unknown	Unknown	Known functional	Indirect	Unknown	Known functional	Unknown	(90)
*M.tb* -MFN1 axis	MFN1-mediated fusion → ATP/OXPHOS ↑ → autophagy ↑	Unknown	Unknown	Unknown	Unknown	Unknown	Known functional	Indirect	Unknown	Indirect	Unknown	(92)
*M.tb* -AMPK/PGC-1α	AMPK → PGC-1α → NRF/TFAM → biogenesis	Unknown	Unknown	Unknown	Unknown	Known functional	Indirect	Indirect	Unknown	Indirect	Unknown	(65)
p62/SQSTM1-MDV axis	p62 KD → TOM20^+^ MDV ↑; MIRO1/2-dependent → RAB7 recruitment	Unknown	TOM20-linked	Unknown	Unknown	Unknown	DRP1/MIRO-linked	Mitophagy flux altered	Known functional	Known functional	Unknown	(135)

A further limitation is definitional: mitophagy is measured in different ways. Some studies infer it from LC3 recruitment, others from changes in mitochondrial protein abundance, mitochondrial acidification dyes, or shifts in mitochondrial membrane potential, and still others from the expression of receptors such as PINK1, Parkin, BNIP3, NIX, and FUNDC1. These readouts do not necessarily distinguish increased mitophagic flux from impaired downstream lysosomal turnover. The distinction matters during mycobacterial infection, where xenophagy and mitophagy share parts of the autophagic machinery, including TBK1 and several cargo adaptors. Competition for that shared machinery may alter bacterial control without reflecting a direct antibacterial function of mitochondrial clearance itself.

Pathway identity also has to be read together with timing, signal intensity, and the metabolic state of the host cell. MtROS is a clear case: controlled mtROS supports inflammatory and antimicrobial responses, whereas excessive or prolonged mtROS promotes mitochondrial damage, necrotic cell death, and bacterial dissemination. The same uncertainty applies to receptor-mediated mitophagy, which cannot yet be assigned a universally protective or pathogenic function. BNIP3-dependent mitophagy favors *M.tb* survival in the models discussed above, while NIX-associated responses have been linked to antimicrobial activity. FUNDC1 is the clearest cautionary example: the available evidence shows compensatory mitophagy in BNIP3-deficient murine macrophages, where further FUNDC1 inhibition increases mtROS and decreases *M.tb* survival. These observations establish context-specific redundancy, but they do not define FUNDC1 function in primary human macrophages.

Pharmacological studies need the same caution. A drug that induces mitophagy and lowers intracellular bacterial burden does not necessarily establish that mitophagy mediates the antibacterial effect. Thiopeptides are a direct illustration, because their mitophagy-inducing and antimycobacterial activities can be separated experimentally. Compounds that alter general autophagy, mitochondrial metabolism, or ROS production may act on several host-defense pathways at once. Future host-directed therapy studies should therefore distinguish target engagement from causal pathway dependence.

Several questions remain open. First, the relative hierarchy and temporal sequence of PINK1-Parkin, BNIP3/NIX, and FUNDC1 activation should be examined within a single infection model rather than inferred across separate studies. Second, receptor-specific mitophagic flux should be measured alongside mtROS, mitochondrial function, inflammatory signaling, xenophagy, and bacterial viability. Third, these pathways need validation in physiologically relevant human macrophages and in the spatially heterogeneous environment of the granuloma and the infected lung. Finally, mitochondrial quality control in adaptive immune cells remains comparatively understudied. Current evidence on mitophagy and MQC in TB remains centered on macrophages, and little is known about other immune and granuloma-associated cell populations, including neutrophils, dendritic cells, NK cells, conventional and γδ T cells, B cells, tissue-resident and foamy macrophages, and multinucleated giant cells. Future studies should determine whether cell-type-specific MQC programs influence antigen presentation, T-cell exhaustion, immunological memory formation, and granuloma maintenance during *M.tb* infection. How these questions are answered will determine whether pathway-selective manipulation of MQC can become a host-directed strategy for TB.

## 6 Conclusion

*M.tb* perturbs mitochondrial homeostasis across proteostasis, stress signaling, dynamics, metabolism, and selective clearance. Current evidence supports a context- and pathway-dependent model. Mitophagy is not uniformly host-protective or pathogen-promoting; its consequences depend on which receptor pathway is engaged, the intensity and timing of mitochondrial stress, metabolic state, and competition with other autophagic processes. However, heterogeneous experimental systems and limited validation in primary human cells and *in vivo* disease settings constrain these conclusions. Receptor-specific mitophagic flux must be defined in matched models and related to xenophagy, mtROS, inflammatory signaling, and bacterial viability before MQC pathways can be targeted for HDT.
